# Molecular epidemiologic investigations of Mycoplasma gallisepticum conjunctivitis in songbirds by random amplified polymorphic DNA analyses.

**DOI:** 10.3201/eid0303.970318

**Published:** 1997

**Authors:** D H Ley, J E Berkhoff, S Levisohn

**Affiliations:** College of Veterinary Medicine, North Carolina State University, Raleigh, North Carolina, USA.

## Abstract

An ongoing outbreak of conjunctivitis in free-ranging house finches (Carpodacus mexicanus) began in 1994 in the eastern United States. Bacterial organisms identified as Mycoplasma gallisepticum (MG) were isolated from lesions of infected birds. MG was also isolated from a blue jay (Cyanocitta cristata) that contracted conjunctivitis after being housed in a cage previously occupied by house finches with conjunctivitis, and from free-ranging American goldfinches (Carduelis tristis) in North Carolina in 1996. To investigate the molecular epidemiology of this outbreak, we produced DNA fingerprints of MG isolates by random amplification of polymorphic DNA (RAPD). We compared MG isolates from songbirds examined from 1994 through 1996 in 11 states, representing three host species, with vaccine and reference strains and with contemporary MG isolates from commercial poultry. All MG isolates from songbirds had RAPD banding patterns identical to each other but different from other strains and isolates tested. These results indicate that the outbreak of MG in songbirds is caused by the same strain, which suggests a single source; the outbreak is not caused by the vaccine or reference strains analyzed; and MG infection has not been shared between songbirds and commercial poultry.


### Molecular Epidemiologic Investigations of *Mycoplasma gallisepticum* Conjunctivitis in Songbirds by Random Amplified Polymorphic DNA Analyses

An ongoing outbreak of conjunctivitis in free-ranging house finches (*Carpodacus mexicanus*) began in 1994 in the eastern United States. Bacterial organisms identified as *Mycoplasma gallisepticum* (MG) were isolated from lesions of infected birds. MG was also isolated from a blue jay (*Cyanocitta cristata*) that contracted conjunctivitis after being housed in a cage previously occupied by house finches with conjunctivitis, and from free-ranging American goldfinches (*Carduelis tristis*) in North Carolina in 1996. To investigate the molecular epidemiology of this outbreak, we produced DNA fingerprints of MG isolates by random amplification of polymorphic DNA (RAPD). We compared MG isolates from songbirds examined from 1994 through 1996 in 11 states, representing three host species, with vaccine and reference strains and with contemporary MG isolates from commercial poultry. All MG isolates from songbirds had RAPD banding patterns identical to each other but different from other strains and isolates tested. These results indicate that the outbreak of MG in songbirds is caused by the same strain, which suggests a single source; the outbreak is not caused by the vaccine or reference strains analyzed; and MG infection has not been shared between songbirds and commercial poultry.

*Mycoplasma gallisepticum* (MG), a well-known cause of diseases of domesticated chickens and turkeys worldwide, most notably causes chronic respiratory disease in chickens and infectious sinusitis in turkeys (1). Although MG is a known pathogen of other gallinaceous birds and has been isolated from ducks and geese, it has not been considered a natural pathogen of wild birds, including songbirds (1). Conjunctivitis in house finches (*Carpodacus mexicanus*) was first reported in February 1994 (2,3). Since then, many ill house finches have been observed at feeders and submitted to wildlife care facilities or veterinary diagnostic laboratories in the Middle Atlantic and Southeastern regions of the United States (2-4). Slow-growing mycoplasmas (mean incubation time = 25 days) were isolated from lesions in clinically ill birds and identified as MG by direct immunofluorescence (3). These findings suggested that MG was the likely cause of this outbreak of conjunctivitis in house finches. MG was also isolated from a blue jay (*Cyanocitta cristata*) that contracted conjunctivitis after being housed in a cage previously occupied by infected house finches (3). This observation suggested that house finches infected with MG may be capable of transmitting the infection horizontally and that other avian species are susceptible to infection and disease. Recently, conjunctivitis has been observed in American goldfinches (*Carduelis tristis*), and we have made two isolates of MG from these birds. This finding suggests that the infection can be transmitted naturally to an additional host species.

To examine the epidemiologic relationships among MG isolates from this outbreak, we used two primer systems previously described for subspecies typing of avian mycoplasmas (5,6) to conduct random amplification of polymorphic DNA (RAPD). MG isolates available for this study represented several presumed epidemiologic relationships: time (1994 through 1996), geographic location (n = 11 states), and host species (n = 3). We compared these isolates with each other, with MG reference and vaccine strains, and with MG isolates from commercial poultry.

### Isolation and Identification of MG Strains

Mycoplasmas were isolated from conjunctival and infraorbital sinus swabs from clinically ill birds (Figure 1) by using Frey's broth medium with 15% swine serum (7). Mycoplasma colonies

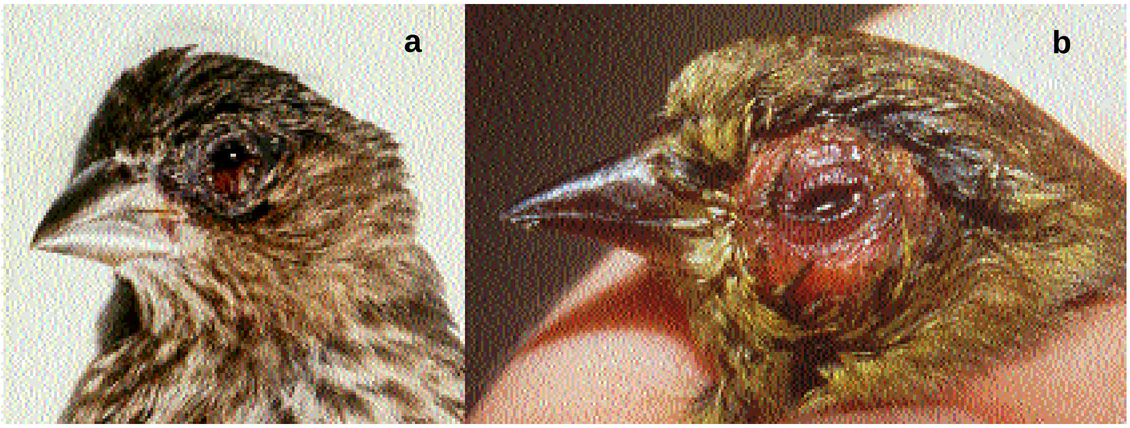

Figure 1. MG isolates have been made from songbirds with clinical signs and gross lesions characterized by mild to severe unilateral or bilateral conjunctival and periorbital swelling with serous to mucopurulent drainage and nasal exudate. Typical gross lesions in a) a female house finch (*Carpodacus mexicanus*) (photo courtesy of D. Earl Green, State of Maryland, Department of Agriculture, College Park, MD) and b) an American goldfinch (*Carduelis tristis*) (photo by K. Joyner, College of Veterinary Medicine, North Carolina State University, Raleigh, NC).

on agar plates were identified as MG by direct immunofluorescence (7), which used fluorescein-conjugated rabbit antiserum provided by S.H. Kleven (Department of Avian Medicine, University of Georgia, Athens, GA). MG strains were isolated from house finches (*C. mexicanus*), American goldfinches (*C. tristis*), and a blue jay (*C. cristata*) with conjunctivitis (Table). Other MG strains analyzed included reference strains S6, R, A5969; vaccine strains F, 6/85 (Intervet Inc., Millsboro, DE), and ts-11 (Select Laboratories, Gainesville, GA); and field isolates from commercial poultry (Table). *Mycoplasma imitans* type strain 4994 was provided by J.M. Bradbury (Department of Veterinary Pathology, University of Liverpool, South Wirral, England).

### Preparation of Mycoplasma DNA

DNA for RAPD analyses was prepared from log phase MG broth cultures. Two to 3 ml of culture containing approximately $10^9$ CFU were centrifuged at 16,000 x g for 6 min, washed two times with phosphate-buffered saline (PBS), and resuspended in 25 µl PBS. The cells were lysed by boiling for 10 min,

Table. *Mycoplasma gallisepticum* isolates from songbirds with conjunctivitis analyzed by random amplification of polymorphic DNA (RAPD)

| Isolate no. | Host species | State of origin | Date isolated (mo/yr) | Isolated by |
|---|---|---|---|---|
| 7994 | House finch | VA | 6/94 | NCSU[a] |
| 11394 | Blue jay | VA | 7/94 | NCSU |
| 16094-1 | House finch | PA | 9/94 | NCSU |
| 16094-5 | House finch | DE | 9/94 | NCSU |
| 16994 | House finch | NC | 9/94 | NCSU |
| 17494 | House finch | DE | 9/94 | NCSU |
| 17694 | House finch | DE | 9/94 | NCSU |
| 17794 | House finch | VA | 9/94 | NCSU |
| K3839 | House finch | MD | 11/94 | SCWDS/UGA[b] |
| 95-11-14C | House finch | NY | 4/95 | UW[c] |
| 13295 | House finch | NC | 8/95 | NCSU |
| K4013 | House finch | PA | 8/95 | SCWDS/UGA |
| K4058 | House finch | GA | 11/95 | SCWDS/UGA |
| K4094 | House finch | TN | 1/96 | SCWDS/UGA |
| 1596-3 | House finch | NC | 2/96 | NCSU |
| 1596-5 | Am. goldfinch | NC | 2/96 | NCSU |
| 1695 | Am. goldfinch | NC | 2/96 | NCSU |
| K4117 | House finch | KY | 2/96 | SCWDS/UGA |
| 1652442 | House finch | MI | 3/96 | MSU[d] |
| K4269 | House finch | OH | 7/96 | SCWDS/UGA |

[a]D. H. Ley, North Carolina State University, College of Veterinary Medicine, Raleigh, NC.
[b]P. Luttrell and J. F. Fischer, Southeastern Cooperative Wildlife Disease Study and S. H. Kleven, Department of Avian Medicine, The University of Georgia, Athens, GA.
[c]C. B. Thomas, School of Veterinary Medicine, University of Wisconsin, Madison, WI.
[d]R. M. Fulton, Animal Health Diagnostic Laboratory, Michigan State University, East Lansing, MI.

placed on ice for 5 to 10 min, and centrifuged at 16,000 x g for 2 min. The resultant supernatant was stored at 4°C for RAPD testing.

### RAPD Analyses

Two previously described RAPD methods (5,6) were used with modification. Method I (5) used a single primer in a total reaction volume of 100 µl, 2.5 units Taq polymerase (Promega, Madison, WI) in the manufacturer's recommended buffer with 2 mM $MgCl_2$, 250 µM each dNTP (Promega), 500 ng primer 1254 (5′CCGCAGCCAA 3′) (Life Technologies, Gaithersburg, MD) and 0.5 µl DNA extract containing 50 to 100 ng DNA. The amplification conditions were four cycles of 94°C for 5 min, 36°C for 5 min, and 72°C for 5 min, followed by 30 cycles of 94°C for 1 min, 36°C for 1 min, and 72°C for 1 min. These were followed by one cycle of 72°C for 10 min.

Method II (6) used three primers (M16SPCR5í = 5′AGGCAGCAGTAGGGAAT 3′, M13F = 5′GTAAAACGACGGC 3′, S1OLIGO 3′ = 5′CATAACTAACATAAGGGCAA 3′) in a total reaction volume of 100 µl, 2.5 units Taq polymerase (Promega) in the manufacturer's recommended buffer with 1.5 mM $MgCl_2$, 250 µM each dNTP (Promega), 500 ng each primer (Life Technologies), and 3.0 µl DNA extract containing 300 to 500 ng DNA. The amplification conditions were three cycles of 94°C for 15 sec, 28°C for 2 min, and 74°C for 3 min, followed by 35 cycles of 94°C for 15 sec, 45°C for 2 min, and 74°C for 3 min. These were followed by one cycle of 72°C for 10 min.

### Gel Electrophoresis

Amplified DNA was separated by electrophoresis in 2% agarose (Pharmacia Biotech AB, Uppsala, Sweden) gels, poststained with ethidium bromide, illuminated with ultraviolet light, and photographed with an FCR-10 camera (Fotodyne Inc., Hartland, WI) and Polaroid 667 film (Polaroid Corp., Cambridge, MA).

Both RAPD methods I (5) and II (6) resulted in DNA banding patterns (Figures 2, 4, and data not shown) that clearly resolved differences among MG vaccine (F, 6/85, ts-11) and reference (S6, R, A5969) strains, thus demonstrating the usefulness of these assays for MG strain identification. Because RAPD analyses differentiated among known MG strains, we were confident that these DNA fingerprinting methods could identify strains. Additionally, RAPD banding patterns of known MG strains constitute the beginning of an MG strain database of DNA fingerprints with which other strains and field isolates can be compared.

RAPD analyses were performed on MG isolates obtained over 2 years from 17 house finches, two American goldfinches, and one blue jay from 11 states in the United States (Table). Upon inspection, all MG isolates from songbirds had essentially identical RAPD banding patterns by either RAPD method, and the patterns differed from those of the reference and vaccine strains tested (Figures 2-5, and data not shown). Therefore, the ongoing outbreak of MG conjunctivitis in songbirds appears to be caused by a single strain or very closely related strains of MG. This suggests the possibility of a single source for the outbreak, probably first involving house finches and more recently American goldfinches, the only two songbird species known to have acquired this disease naturally. These findings demonstrate that the MG strain involved is not host-species specific under natural conditions. Infection in the blue jay was apparently nosocomial, most likely resulting from exposure to MG-infected house finches or fomites while the birds were housed at a wildlife care facility. Isolation of what appears to be the same strain of MG from a blue jay exposed to infected house finches demonstrates the potential for this outbreak to spread to additional host species.

MG isolates from songbirds had RAPD banding patterns that differed from *M. imitans* (Figure 4, and data not shown). *M. imitans*, isolated from wild birds (duck, goose, and partridge) in Europe, cross-reacts with MG by immunofluorescence and growth inhibition tests but has only approximately 40% to 46% genetic homology with MG (type strain PG31) by DNA-DNA hybridization (8). Therefore, to rule out the possibility that isolates identified as MG by immunofluorescence tests were not *M. imitans*, we compared the RAPD banding pattern of the *M. imitans* strain: it was markedly different from that of the finch isolates and other MG strains tested (Figure 4, and data not shown). In addition, we confirmed the finch isolates as MG (2,3) by using a commercially available MG-specific polymerase chain reaction-based test (FlockChek MG DNA Probe, IDEXX Laboratories, Inc., Westbrook, ME). Differential diagnoses of conjunctivitis in songbirds should also include the

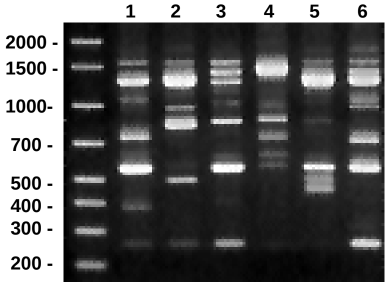

Figure 2. RAPD (method I) patterns of MG vaccine (lanes 1-3) and reference (lanes 4-6) strains. DNA base pair size standards are shown on the left. Lane 1 = ts-11; lane 2 = F; lane 3 = 6/85; lane 4 = R; lane 5 = S6; and lane 6 = A5969. Use of RAPD method I on these MG strains resulted in unique banding patterns that can be easily distinguished from one another.

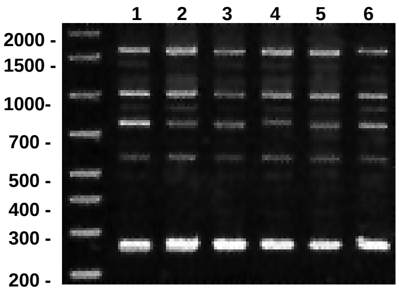

Figure 3. RAPD (method I) patterns of MG isolates from songbirds. DNA base pair size standards are shown on the left. Lane 1 = 7994 (house finch); lane 2 = 11394 (blue jay); lane 3 = 16094-1 (house finch); lane 4 = 16994 (house finch); lane 5 = 13295 (house finch); and lane 6 = 1596-6 (American goldfinch).

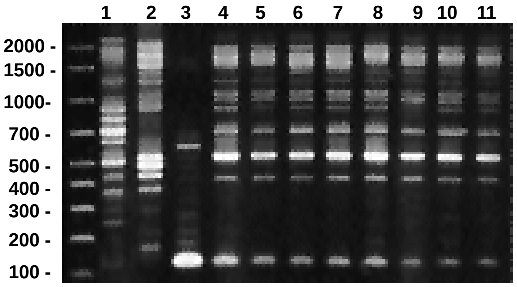

Figure 4. RAPD (method II) patterns of MG vaccine strains (lanes 1-2) and isolates from house finches (lanes 4-11), and *M. imitans* type strain (lane 3). DNA base pair size standards are shown on the left. Lane 1 = ts-11; lane 2 = 6/85; lane 3 = *M. imitans*; lane 4 = K3839; lane 5 = K4013; lane 6 = K4013; lane 7 = K4117; lane 8 = 7994; lane 9 = 1652442; lane 10 = K4058; lane 11 = K4269. An additional RAPD primer set (method II) was used to determine whether method I accurately determined MG strain identities.

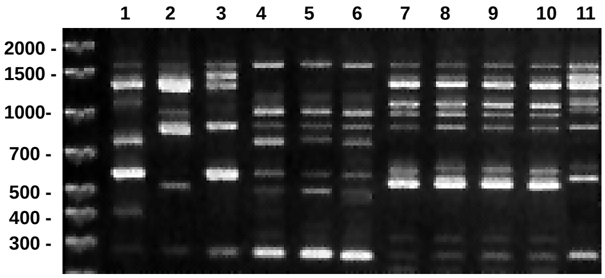

Figure 5. RAPD (method I) patterns of MG vaccine strains (lanes 1-3), isolates from songbirds (lanes 4-6), and isolates from commercial poultry (lanes 7-11). DNA base pair size standards are shown on the left. Lane 1 = ts-11; lane 2 = F; lane 3 = 6/85; lane 4 = 7994 (house finch); lane 5 = 17794 (house finch); lane 6 = 1596 (American goldfinch); lanes 7-10 = separate isolates made from commercial turkeys; lane 11 = isolate from commercial chickens.

newly characterized *Mycoplasma sturni* recovered from a European starling (*Sturnus vulgaris*) and a mockingbird (*Mimus polyglottos*) in Connecticut (9,10). We have recovered *M. sturni* from conjunctival swabs collected from one blue jay and six mockingbirds from Florida (Ley, Berkhoff, Levisohn, unpub. obs.) However, there is no evidence that either *M. imitans* or *M. sturni* is involved in the present epidemic of conjunctivitis in American goldfinches and house finches. The possible diagnostic complications that these *Mycoplasma* spp. represent should not be ignored.

Figure 5 shows RAPD banding patterns (method I) of MG vaccine strains and isolates from songbirds and commercial poultry. The poultry isolates include four made in 1996 from an outbreak in Missouri turkeys and one made in 1994 from North Carolina chickens. The MG isolates tested from commercial turkeys had RAPD banding patterns essentially identical to each other, which would be the result if a single or dominant strain were responsible for the outbreak. The isolate from commercial chickens appears similar to that from turkeys, and isolates from both are clearly different from vaccine strains. As previously observed (Figures 2-4), songbird MG RAPD banding patterns were essentially identical to each other and clearly different from the vaccine strains. Figure 5 further shows that the songbird MG RAPD banding pattern was different from the patterns of isolates from chickens and turkeys. This initial, limited comparison of isolates from poultry and songbirds provided no evidence of shared MG strains. We are now applying RAPD analyses to additional MG isolates, both contemporary and archival, from commercial poultry to search for MG strains shared by songbirds and poultry or other birds.

RAPD method I was used to conduct routine screening and most comparative tests because of its reproducibility, discriminating capability, and ease of interpretation; however, the use of two different RAPD primer systems is valuable for confirming apparent relationships. In our study, a second RAPD primer set (method II) was used to confirm the MG strain identities determined by method I. Even though RAPD method II generally resulted in more bands, MG isolates from songbirds had essentially identical DNA fingerprints (Figure 4, and data not shown), thus supporting results by RAPD method I.

We standardized sample preparation and gel electrophoresis protocols to ensure reproducibility of RAPD results. Further standardization of technique and implementation of a computer-based gel analysis system should allow for comparison of results between laboratories, thus enhancing the usefulness and power of these procedures. We have found that RAPD analyses are useful for MG strain identification and molecular epidemiologic investigations. Applying this technology to the current epidemic of conjunctivitis in songbirds showed that all MG isolates from songbirds had essentially identical RAPD banding patterns to each other, but different patterns from all other strains and isolates tested. These results indicated that 1) the outbreak of MG in songbirds is caused by the same strain, suggesting a single source; 2) the outbreak is not caused by the vaccine or reference strains analyzed; and 3) MG infection does not appear to have been shared between songbirds and commercial poultry.

Fischer et al. (4) have stated that the epidemic of MG conjunctivitis in songbirds parallels emerging human diseases. However, even though this outbreak has no direct effect on humans (no evidence exists that MG causes zoonotic infections), the public has been aware of and involved in this epidemic, as observers (and feeders) of songbirds and as participants (citizen scientists) in such surveys as the House Finch Disease Survey (Project FeederWatch, Cornell Lab of Ornithology, PO Box 11, Ithaca, NY 14851-0011, and http://www.ornith.cornell.edu/CS/HOFI/main.html). Ultimately, the effects of this epidemic depend on its evolving course, the avian host species involved, the degrees to which the species are affected, and the species' relationships to the public's concerns (e.g., scientific, economic, recreational, professional). RAPD analyses of additional MG isolates from our archives and those of others and continued analysis of contemporary MG isolates from songbirds and other birds including commercial poultry will provide an exceptional opportunity to track this emerging disease.

### Acknowledgments

We thank Chris York, Douglas Anderson, Gina Neri, Rebecca Packer, and Tyler Lund for valuable technical assistance; L. Degernes, K. Joyner, and L. Powers for their cooperation and submission of specimens; The Wildlife Center of Virginia; Tri-State Bird Rescue and Research, Inc.; New York State Department of Environmental Conservation; Southeastern Cooperative Wildlife Disease Study and Department of Avian Medicine, University of Georgia; School of Veterinary Medicine, University of Wisconsin; and Animal Health Diagnostic Laboratory, Michigan State University. Resources to support this research were provided by the State of North Carolina and a grant from the U. S. Poultry and Egg Association.

**David H. Ley,* J. Edward Berkhoff,* and Sharon Levisohn†**

*College of Veterinary Medicine, North Carolina State University, Raleigh, North Carolina, USA; †Visiting Research Scientist, Kimron Veterinary Institute, Bet Dagan, Israel

### References

1. Yoder HW Jr. *Mycoplasma gallisepticum* infection. In: Hofstad MS, Barnes HJ, Calnek BW, Reid WM, Yoder HW Jr, editors. Diseases of poultry. 9th ed. Ames (IA): Iowa State University Press; 1991. p. 198-212.
2. Luttrell MP, Fischer JR, Stallknecht DE, Kleven SH. Field investigation of *Mycoplasma gallisepticum* infections in house finches (*Carpodacus mexicanus*) from Maryland and Georgia. Avian Dis 1996;40:335-41.
3. Ley DH, Berkhoff JE, McLaren JM. *Mycoplasma gallisepticum* isolated from house finches (*Carpodacus mexicanus*) with conjunctivitis. Avian Dis 1996;40:480-3.
4. Fischer JR, Stallknecht M, Luttrell P, Dhondt AA, Converse KA. Mycoplasmal conjunctivitis in wild songbirds: the spread of a new contagious disease in a mobile host population. Emerg Infect Dis 1997;3:69-72.

5. Geary SJ, Forsyth MH, Aboul Saoud S, Wang G, Berg DE, Berg CM. *Mycoplasma gallisepticum* strain differentiation by arbitrary primer PCR (RAPD) fingerprinting. Mol Cell Probes 1994;8:311-6.
6. Fan HH, Kleven SH, Jackwood MW. Application of polymerase chain reaction with arbitrary primers to strain identification of *Mycoplasma gallisepticum*. Avian Dis 1995;39:729-35.
7. Kleven SH, Yoder HW Jr. Mycoplasmosis. In: Purchase HG, Arp LH, Domermuth CH, Pearson JE, editors. A laboratory manual for the isolation and identification of avian pathogens. 3rd ed. Kennett Square (PA): American Association of Avian Pathologists; 1989. p. 57-62.
8. Bradbury JM, Abdul-Wahab OM, Yavari CA, Dupiellet JP, Bove JM. *Mycoplasma imitans* sp. nov. is related to *Mycoplasma gallisepticum* and found in birds. Int J Syst Bacteriol 1993;43:721-8.
9. Forsyth MH, Tully JG, Gorton TS, Hinckley L, Frasca SJ, Van Kruiningen J, et al. *Mycoplasma sturni* sp. nov., from the conjunctiva of a European starling (*Sturnus vulgaris*). Int J Syst Bacteriol 1996;46:716-9.
10. Frasca SJ, Hinckley L, Forsyth MH, Groton TS, Geary SJ, Van Kruiningen HJ. Mycoplasmal conjunctivitis in a European Starling. J Wildl Dis 1997;33:336-9.
